# Correlations between chest-CT and laboratory parameters in SARS-CoV-2 pneumonia: A single-center study from Italy

**DOI:** 10.1097/MD.0000000000025310

**Published:** 2021-04-09

**Authors:** Antonio Orlacchio, Fulvio Gasparrini, Silvia Roma, Matteo Santangelo Ravà, Eva Salvatori, Daniele Morosetti, Elsa Cossu, Jacopo Maria Legramante, Carla Paganelli, Sergio Bernardini, Marilena Minieri

**Affiliations:** a Radiology, Department of Emergency, Tor Vergata University Hospital, Department of Surgical Science, University of Rome “Tor Vergata”, Rome; b Department of Diagnostic and Interventional Radiology, General Hospital, Frosinone, Italy; c Bergen Center for Ethics and Priority Setting, University of Bergen, Bergen, Norway; d Department of Emergency, Tor Vergata University Hospital, Department of Systems Medicine; e Department of Emergency; f Departments of Experimental Medicine and Laboratory Medicine, Tor Vergata University Hospital, University of Rome “Tor Vergata”, Rome, Italy.

**Keywords:** biochemistry, biomarkers, blood cell count, COVID-19, reverse transcriptase polymerase chain reaction, SARS-CoV-2

## Abstract

To investigate the relationship between damaged lung assessed by chest computed tomography (CT) scan and laboratory biochemical parameters with the aim of finding other diagnostic tools.

Patients who underwent chest CT for suspected Corona Virus Disease-2019 (COVID-19) pneumonia at the emergency department admission in the first phase of COVID-19 epidemic in Italy were retrospectively analyzed. Patients with both negative chest CT and absence of the novel coronavirus in nasopharyngeal or oropharyngeal real-time reverse transcriptase polymerase chain reaction (RT-PCR) swabs were excluded from the study. A total of 462 patients with positive CT scans for interstitial pneumonia were included in the study (250 males and 212 females, mean age 57 ± 17 years, range 18–89). Of these, 344 were positive to RT-PCR test, 118 were negative to double RT-PCR tests.

CTs were analyzed for quantification of affected lung volume visually and by dedicated software. Statistical analysis to evaluate the relationship between laboratory analyses and CT patterns and amount of damaged lung related with COVID-19 pneumonia was performed in 2 groups of patients: positive RT-PCR COVID-19 group and negative RT-PCR COVID-19 group, but both with positive CT scans for interstitial pneumonia.

Lymphocytopenia, C-reactive protein (CRP), lactate dehydrogenase (LDH), d-dimer, and fibrinogen increased levels occurred in most patients without statistically significant differences between the 2 groups with CT scans suggestive for COVID-19. In fact, in both groups the volume of lung damage was strongly associated with altered laboratory test results, even for patients with negative RT-PCR test.

The decreased number of lymphocytes, and the increased levels of CRP, LDH, d-dimer, and fibrinogen levels are associated with SARS-CoV 2 related pneumonia. This may be useful as an additional diagnostic tool in patients with double negative RT-PCR assay and with highly suspected clinic and chest CT features for COVID-19 to isolate patients in a pandemic period.

## 1 Introduction

After the first case of pneumonia caused by a novel coronavirus in December 2019 in Wuhan (Hubei, China), the disease has shown a rapid diffusion and a huge aggressiveness. On January 30th, 2020, the World Health Organization (WHO) declared Corona Virus Disease-2019 (COVID-19) as a public international concern,^[1]^ and on March 12th, as a consequence of the increasing number of cases, the WHO declared COVID-19 as a pandemic disease.^[2]^

On January 31st, the Italian Government declared the state of emergency in the country.

As of April 25th, in Italy 199.414 infected patients were reported, with 26.977 deaths and 66.624 recovered.^[3]^

According to the guidelines, patients suspected of COVID-19 must be subjected to swab analysis and, if necessary, to chest imaging. Namely, the chest radiography is offered as first step and supplementary computed tomography (CT) in more severe cases or in case of discrepancy between clinical and radiographic characteristics.^[4]^

Several studies have reported that at initial presentation real-time reverse transcriptase polymerase chain reaction (RT-PCR) method applied to respiratory tract specimens has a sensitivity that ranges between 60% and 70% due to intrinsic limitations like viral load in different anatomic sites, sampling procedures, and technical reasons (reagents, sample transport condition, etc).^[5–7]^

Some patients with positive chest CT findings for interstitial pneumonia may present negative RT-PCR test even after double-swab analysis. This condition complicates the management of patients suspected of COVID-19 and the emergency department flow.

Recently, some authors reported an outline of the most representative laboratory abnormalities found in patients with COVID-19 infection.^[8,9]^

The aim of the present study was to investigate chest CT features of patients positive for COVID-19 by RT-PCR swab in comparison to patients negative to double-swab test but with interstitial pneumonia by CT scan images. The diagnostic performance of chest CTs along with laboratory abnormalities could be useful in investigating other diagnostic tools in patients with highly suspicious clinical symptoms and CT scans typical for COVID-19 but negative to the RT-PCR swab test.

## 2 Materials and methods

### 2.1 Study population

This retrospective study was performed at the Covid4 hospital in Rome, Italy and was approved by the local Ethics Committee, and the need for the informed consent was waived by the institutional review board.

The study included consecutive symptomatic patients with suspected COVID-19 interstitial pneumonia who underwent chest CT at emergency department admission from March 6th to April 25th, 2020. Laboratory findings of each patient were recorded at admission. CT was performed within 12 hours from the clinical evaluation and laboratory findings.

In order to select chest CT scans for analysis, our exclusion criteria were:

(a) patient without nasopharyngeal swab;
(b) negative chest CT or severe motion artifact on chest CT;
(c) patient with any other laboratory test positive for other viral or bacterial infection.

Patients included in the study were categorized in 2 groups: patients with highly suspicious CT and positive RT-PCR (group 1) versus patients with highly suspicious CT and double negative RT-PCR swab test (group 2).

Laboratory parameters of each group performed the day of admission were retrospectively investigated.

### 2.2 CT protocol

Non-enhanced chest CT scan was performed in supine position, during inspiratory breath-hold, from the apex to the lung bases, with a multidetector scanner (CT Evolution EVO; GE Healthcare, Chalfont St. Giles, Buckinghamshire, UK). Low-dose CT acquisition was executed as follows: tube voltage, 120 kV; automatic tube current control (40–90 mAs) was used; pitch, 1; collimation, 0.625 mm. Image data sets were reconstructed with 1.25-mm slice thickness.

### 2.3 Analysis of CT images

Two radiologists with 15 and 7 years of thoracic imaging experience, respectively, blinded to the clinical data and laboratory findings reviewed the CT images independently and resolved discrepancies by consensus. All images were viewed on both lung (width, 1500 Hounsfield Unit [HU]; level, −700 HU) and mediastinal (width, 350 HU; level, 40 HU) settings. The presence or absence of following features was recorded: ground-glass opacities (GGO), consolidation (CO), traction bronchiectasis, bronchial wall thickening, crazy paving (CP), subpleural bands, and lesion distribution.

The number of involved lobes was registered. The prevalence in the upper (above the level of the carina), middle (between carina and infrapulmonary vein), or lower zone (below the level of the infrapulmonary vein) was recorded. The axial distribution was classified as peripheral (prevalent in the outer third of the lung) or central (predominant in the inner two-third). The distribution pattern was classified as diffuse when a clear predominant cranio-caudal or axial distribution was absent.

**Figure 1:**
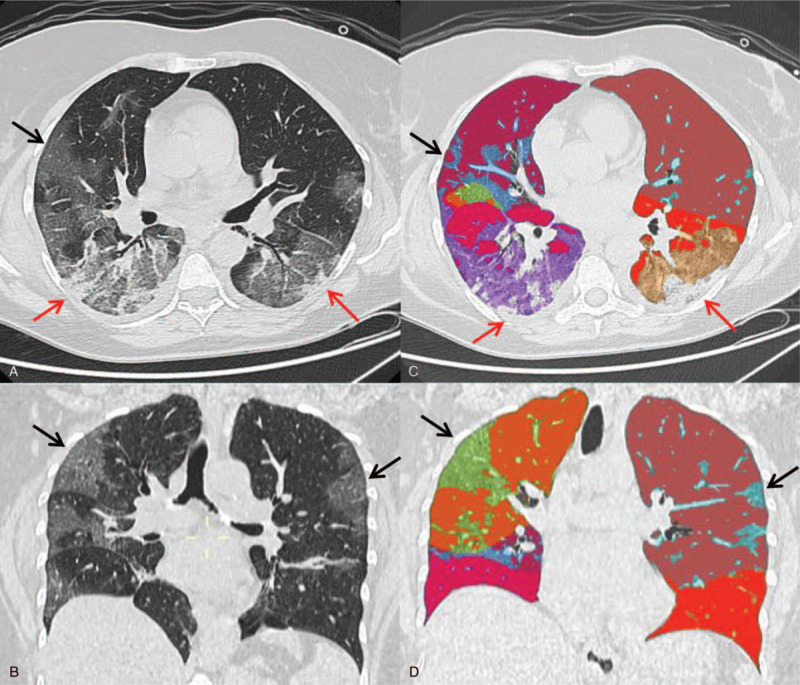
(A) Axial and (B) coronal images of chest CT. (C and D) Reformation with IntelliSpace COPD software used to calculate ground-glass volume in 57 y.o. Male patient with negative RT-PCR nasopharyngeal swab. CT images show bilateral patchy ground-glass opacities (black arrows) with peripheral consolidation (red arrows). COPD software calculated 32% of ground-glass patterns. COPD = chronic obstructive pulmonary disease, RT-PCR = real-time reverse transcriptase polymerase chain reaction.

In addition, a semi-automatic image processing software was used to calculate the well-aerated lung volume, ground-glass volume, and consolidation in each patient (Fig. 1).

The software-based evaluation of the altered lung parenchyma was performed on a dedicated workstation using the extension IntelliSpace Portal 7.0 (Philips, UK).

**Figure 2:**
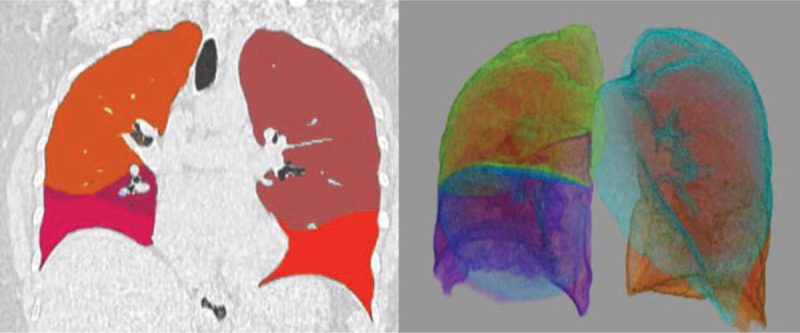
Patient aged 65 with positive RT-PCR for SARS-CoV-2 infection. Coronal chest CT images were obtained with a semi-automatic segmentation of lung parenchyma performed using COPD IntelliSpace Portal 7.0 software. The algorithm provides a color map of different lobes and segments displayed as multiplanar reconstruction. In this patient, images show a well-aerated lungs parenchyma depicted as uniform distribution of colors. COPD = chronic obstructive pulmonary disease, RT-PCR = real-time reverse transcriptase polymerase chain reaction.

A semi-automatic lung segmentation and analysis of lung parenchyma was obtained using the CT-chronic obstructive pulmonary disease (COPD) tool. In case of unsatisfactory lung segmentation, the user amended the lung contours with a manual tool. The definition of normal lung by software segmentation was determined by density references from the literature,^[10]^ namely in the interval between −950 HU and −700 HU. Ground-glass volume was determined in the range between −700 HU and −300 HU and the consolidated parenchyma volume was considered in the range between −300 HU and 40 HU. Furthermore, using the overall lung volume provided by software, the absolute volume of the altered lung volume was calculated (Fig. 2).

### 2.4 Analysis of laboratory findings

RT-PCR analyses were performed by CFX96 Touch Real-Time PCR Detection System (Bio-Rad Laboratories Inc.) with Allplex SARS-CoV-2 assay (Seegene Inc., South Korea).

RT-PCR results were retrospectively evaluated in all patients: patients with only 1 positive nasopharyngeal swab were defined as “positive” and patients with 2 or more negative swabs as “negative.”

The following laboratory abnormalities on blood tests on admission were also considered: lymphocytopenia (defined as lymphocyte count < 1.1 × 10^3^/μL); reduction (<150 × 10^3^/μL) of platelet count; increased (>0.50 mg/dL) C-reactive protein (CRP) levels; increased (>220 U/L) lactate dehydrogenase (LDH); increased (>500 ng/mL) d-dimer; increased (>400 mg/dL) fibrinogen.

### 2.5 Statistical analysis

We created 3 variables to measure damage to the lungs. The first variable is the percentage of lungs that are not compromised (well-aerated volume). This was computed using the volume −700 HU from the software COPD and then we calculated the compromised volume. The second variable is the finding of damaged lung (ordinal). We assigned to patients a value from 1 to 5 representing <10%, 10% to 25%, 25% to 50%, 50% to 75%, >75% of lung segments damaged, based on the examination of the chest CT scan using the classification of European Society of Radiology – European Society of Thoracic Imaging (ESR-ESTI).^[10]^

Finally, we created a binary variable that indicates when 15 or more segments of the lungs were affected (i.e., highly damaged); this coincides with the last category of the ordinal variable.

We performed *t* tests to compare the prevalence of different patterns among the group of RT-PCR positive and negative COVID-patients.

We performed logistic regression for the continuous variable for pulmonary damage and K Pearson's Chi squared test for the ordinal variable and for the binary variable for pulmonary damage to analyze the association between the biochemical parameters and the damage revealed in the CT scan. We included an interaction term in the logistic regression using the COVID-19 RT-PCR test result positive to study whether the relation between the laboratory exams and CT scan was dependent on the COVID-19 test result. All statistical analyses were conducted using Stata 14.0 (STATA, College Station, TX).

We analyzed the percentage of the alteration of laboratory parameters, prevalence of patterns, and means of the damaged quantification, for the whole sample and for the group positive to RT-PCR test and negative to RT-PCR. We used statistical tests giving more constant results. The value for the whole sample and the value that resulted differently with a *t* test for a *P*-value of .05 between the 2 groups were reported.

## 3 Results

### 3.1 Patient population

A total of 993 CT exams were performed during the study period in patients with suspected COVID-19 symptoms (cough, fever, dyspnea).

From the total, 101 patients were excluded due to lack of RT-PCR results or incomplete laboratory data; 415 patients were excluded as CT scans were negative for interstitial pneumonia. Four hundred seventy seven patients had positive CT scans suspected for SARS-CoV-2 interstitial pneumonia. Of these, 344 had positive RT-PCR test for COVID-19 diagnosis.

Of the 133 double-swab test negatives, 15 patients who tested positive for other etiological agents responsible for interstitial pneumonia were excluded. A total of 462 patients were included in the study (250 males and 212 females; mean age 57 ± 17 yrs, range 18–89).

All patients were tested for the presence of other viruses (i.e., respiratory syncytial virus, metapneumovirus, coronavirus, rhino/enteroviruses, parainfluenza virus, etc). All tests were negative (data not shown).

### 3.2 Computed tomography findings

Using RT-PCR as reference standard, sensitivity, specificity, and accuracy of chest CT for diagnosis of COVID-19 pneumonia were 92% (95% confidence interval [CI], 85%–96%), 62% (95% CI, 56%–68%), and 78% (95% CI 73%–82%), respectively.

In the 2 groups, positive RT-PCR and negative RT-PCR, we have found no statistical difference in lung damages.

Pulmonary changes related to viral infection were bilateral in 92.2% of cases, GGO was identified in 98% of cases, traction bronchiectasis in 5.8%, CP in 36.9% of patients, consolidation in 68.9%, and subpleural bands in 29.1% of the cases.

No statistically significance differences were found in the extent of the different lung damages in the 2 groups, positive RT-PCR and negative RT-PCR.

Looking at the quantification of the damage in the lungs, the percentage of affected lung was 30% in both groups with an average GGO volume of 664 cm^3^ in the negative RT-PCR group and 795 cm^3^ in the positive RT-PCR group; the *t* test confirms this difference to not be significant (*P*-value .186). The average number of sections affected was 10 and we found no significant difference (*P*-value .121) looking into the RT-PCR positive and negative (Tables 1 and 2).

**Table 1:** Computed tomography findings analyzed by *t* test for mean difference between the 2 groups, positive RT-PCR and negative RT-PCR.

Description	Overall	Negative RT-PCR	Positive RT-PCR	(1) vs (2)	*P*-value *t* test
Bilateral	0.922	0.927	0.917	0.011	.843
Ground glass	0.981	0.982	0.979	0.003	.923
Consolidation	0.689	0.764	0.604	0.159	.083^∗^
Ground glass and consolidation	0.670	0.745	0.583	0.162	.082^∗^
Traction bronchiectasis	0.058	0.036	0.083	−0.047	.315
Bronchial wall	0.233	0.182	0.292	−0.110	.192
Crazy paving	0.369	0.327	0.417	−0.089	.353
Subpleural	0.291	0.218	0.375	−0.157	.082^∗^
Upper coronal distribution	0.583	0.491	0.688	−0.197	.044^∗∗^
Middle coronal distribution	0.777	0.727	0.833	−0.106	.201
Lower coronal distribution	0.922	0.927	0.917	0.011	.843
Central axial distribution	0.019	0.036	0.000	0.036	.186
Peripheral axial distribution	0.583	0.600	0.562	0.038	.704
Diffuse axial distribution	0.398	0.364	0.438	−0.074	.450

Distribution of lung alterations in positive RT-PCR patients and negative RT-PCR patients: upper coronal distribution: above the level of the carina; middle coronal distribution: between carina and infrapulmonary veins; lower coronal distribution: below the level of the infrapulmonary veins. Central axial distribution: predominant in the inner two-third of the lungs; peripheral axial distribution: prevalent in the outer third of the lungs; diffuse axial distribution: absence of a predominant distribution of CT alterations in the lungs. RT-PCR = real-time reverse transcriptase polymerase chain reaction.

∗ *P* < .1.

∗∗ *P* < .05. ^∗∗∗^*P* < .01.

**Table 2:** Pulmonary damage quantification analyzed by *t* test for mean difference between the 2 groups, positive RT-PCR, and negative RT-PCR.

Description	Overall	Negative RT-PCR	Positive RT-PCR	(1) vs (2)	*P*-value *t* test
Total right segments	5.408	5.055	5.812	−0.758	.279
Total left segments	4.631	4.109	5.229	−1.12	.039^∗∗^
Total segments	10.039	9.164	11.042	−1.878	.121
Ground-glass volume	725.314	664.167	795.377	−131.21	.186
Affected lungs percentage	30.40%	30.60%	30.20%	0.004	.928
Less than 10%	0.120	0.111	0.130	−0.019	.770
Between 10% and 25%	0.160	0.259	0.043	0.216	.003^∗∗∗^
Between 25% and 50%	0.220	0.204	0.239	−0.035	.674
Between 50% and 75%	0.110	0.093	0.130	−0.038	.551
More than 75%	0.390	0.333	0.457	−0.123	.212
Interested volume numeric	3.490	3.278	3.739	−0.461	.112

RT-PCR = real-time reverse transcriptase polymerase chain reaction. ^∗^*P* < .1.

∗∗ *P* < .05.

∗∗∗ *P* < .01.

### 3.3 Laboratory tests

In positive RT-PCR patients, lymphocytopenia was present in 85% of cases, CRP elevation in 85%, and LDH elevation in 77%, d-dimer and fibrinogen, respectively, in 77% and 89%, respectively.

In negative RT-PCR patients, lymphocytopenia was present in 72% of cases, CRP elevation in 88%, and LDH elevation in 78%, d-dimer and fibrinogen in 85% and 92, respectively.

No statistically significant differences in those findings were found in the 2 groups (Table 3).

**Table 3:** Laboratory exams outcomes by *t* test for mean difference between the 2 groups, positive RT-PCR and negative RT-PCR.

Description	Overall	Negative RT-PCR	Positive RT-PCR	(1) vs (2)	*P*-value *t* test
Lymphocytes	0.786	0.727	0.854	−0.127	.119
Platelets	0.155	0.145	0.167	−0.021	.770
CRP	0.873	0.889	0.854	0.035	.604
LDH	0.777	0.782	0.771	0.011	.895
d-Dimer	0.814	0.857	0.771	0.086	.279
Fibrinogen	0.911	0.925	0.896	0.029	.617

CRP = C-reactive protein, LDH = lactate dehydrogenase, RT-PCR = real-time reverse transcriptase polymerase chain reaction. ^∗^*P* < .1. ^∗∗^*P* < .05. ^∗∗∗^*P* < .01.

### 3.4 Chest CT-laboratory tests correlation

Overall, a greater proportion of damaged lung is associated with a positive test for all laboratory tests. The strongest correlation is between lymphocytopenia, d-dimer increase and increased damage of the lungs on chest CT exam. Positive, significant correlation was also identified in the LDH, CRP, and fibrinogen tests.

For the ordinal and the binary lung damage variables, we found a correlation for the whole sample (RT-PCR COVID-19 positive and negative), which was significant for lymphocytopenia, CRP, LDH, d-dimer, and fibrinogen. For our continuous variable (affected lung percentage), we found a positive correlation with lymphocytopenia (*P*-value .023), and d-dimer (*P*-value .006) using the logistic regression. When we introduced the interaction term to assess whether the association between lymphocyte test result and lung damage differed for those who tested positive for COVID-19 using RT-PCR, we found that the coefficient of the continuous variable (proportion of lung affected) was still significant but the interaction term was not significant. Our interpretation is that the RT-PCR status does not affect the link between the damage measured and the laboratory tests. We introduced the interaction terms also in the logistic regression with the other laboratory tests but all coefficients were not significant, perhaps for the small sample of population.

When we stratified the analysis by COVID-19 RT-PCR test results, we found similar associations between laboratory results and lung damage for both groups analyzed. While the associations between laboratory tests and lung damage were stronger among the sample of COVID-19 RT-PCR positive patients compared to the COVID-19 RT-PCR negative patients, the volume of lung damage was still strongly associated with altered laboratory test results, even for RT-PCR negative patients. More in detail, looking at lymphocytopenia we found K Pearson's Chi squared test to be significant in both groups analyzed and the same evidence was for d-dimer. This confirms how similarly the 2 groups behave. Patients with lung damage larger than 75% and the RT-PCR positive tests had significant correlation with other altered laboratory test results (Table 4). Besides, we found a similar association between the 2 groups for the anomalous fibrinogen level.

**Table 4:** Evaluation between lung damage and laboratory test outcomes in the whole sample and group negative RT-PCR and positive RT-PCR groups by logistic regression for continuous variable and Pearson's Chi squared test (*P*-val) for binary and ordinal variables.

Sample tested	Whole sample			Negative RT-PCR		Positive RT-PCR	
Variable tested	Affected lung percentage	Interested volume cat^[1–5]^	Lung damage more than 75%	Interested volume cat^[1–5]^	Lung damage more than 75%	Interested volume cat^[1–5]^	Lung damage more than 75%
Lab test	Logit reg. (*P*-val)	Pearson's Chi squared test (*P*-val)	Pearson's Chi squared test (*P*-val)	Pearson's Chi squared test (*P*-val)	Pearson's Chi squared test (*P*-val)	Pearson's Chi squared test (*P*-val)	Pearson's Chi squared test (*P*-val)
Lymphocytopenia	.023^∗∗^	<.001^∗∗∗^	.002^∗∗∗^	.003^∗∗∗^	.061^∗^	.001^∗∗∗^	.012^∗∗^
Fibrinogen	.207	<.001^∗∗∗^	.015^∗∗^	.054^∗^	.153	.006^∗∗∗^	.037^∗∗^
CRP	.122	.009^∗∗∗^	.015^∗^	.081^∗^	.066^∗^	.029^∗∗^	.089^∗^
LDH	.070^∗^	.054^∗^	.005^∗∗^	.249	.042^∗∗^	.064^∗^	.052^∗^
d-Dimer	.006^∗∗∗^	<.001^∗∗∗^	<.001^∗∗∗^	.001^∗∗∗^	.047^∗∗^	<.001^∗∗∗^	.001^∗∗∗^

CRP = C-reactive protein, LDH = lactate dehydrogenase, RT-PCR = real-time reverse transcriptase polymerase chain reaction.

∗ *P* < .1.

∗∗ *P* < .05.

∗∗∗ *P* < .01.

## 4 Discussion

The continually increasing number of suspected COVID-19 cases is overwhelming medical staff.

An early diagnosis of COVID-19 is essential both for the patient's prognosis and for reducing the spread of the virus.

According to the guidelines,^[11]^ RT-PCR assay on upper or lower respiratory tract specimens is the “gold standard” of clinical diagnosis being relatively fast and easy to carry out in hospital laboratories. However, the gold standard also has limitations,^[12]^ with a RT-PCR sensitivity rate reported to be around 60%.^[5]^

Kucirka et al have shown an inverse correlation between the number of false negatives and the sampling timing (different period of the disease development) with a median false negative rate of 39% on the day of symptom onset, evaluating the accuracy of different respiratory specimens in the laboratory diagnosis and monitoring the viral shedding of SARS-CoV-2 infections.^[13]^

The problem of false-negative results of the RT-PCR test creates a group of patients without a definitive diagnosis, difficult to manage.

Ai et al found 308 of 1014 patients with suspected CT findings for COVID-19 pneumonia and negative RT-PCR samples; of these 147 patients were considered as highly likely cases, considering the clinical characteristics.^[14]^

In the present study, 118 patients had a positive chest CT scan with double negative RT-PCR.

Several studies have shown alterations of some laboratory parameters with greater frequency in patients with COVID-19, such as lymphocyte count, CRP, LDH, d-dimer, and fibrinogen.^[7,15,16]^

Based on these considerations, in this retrospective study we analyzed correlation between CT alterations and laboratory parameters to find another diagnostic tool to associate with CT and RT-PCR tests in the diagnosis and management of highly suspicious patients of SARS-CoV-2 infection. In the current study, we found no statistically significant differences in some laboratory tests between RT-PCR positive patients and RT-PCR negative patients with typical findings on chest CT for Sars-CoV-2 pneumonia. Our results suggest that patients with suspected CT findings for COVID-19 pneumonia and abnormal laboratory analyses, usually found in COVID-19, and negative swab test could be, very likely, RT-PCR false negatives.

No difference among male and female results was found (data not shown).

CT has a pivotal role for diagnosis and monitoring the care of patients with COVID-19 pneumonia. In this retrospective study, the sensitivity of chest CT for COVID-19 pneumonia was 91% (95% CI, 85–96%), greater than that reported for RT-PCR test, in accordance with other studies.^[14]^

Given its high sensitivity, CT has already been adopted as a diagnostic criterion for COVID-19 in the revised 5th edition of the Guideline of Diagnosis and Treatment in Hubei Province, China.^[17]^

Also, the Fleischner Society suggests that diagnosis may be presumed based on CT findings in patients with moderate to severe characteristics of COVID-19 even in the absence of test positivity.^[18,19]^

A limitation of CT is the possibility of having some false positive cases because the CT imaging features of COVID-19 pneumonia are like those of other viral pneumonia. However, the evaluation of clinical symptoms and laboratory biochemical parameters can reduce the possibility of false positivity.

However, this study had some limitations. It is a single-center, retrospective study with small size of sample population. We do not report patients’ outcomes, because not available yet. Furthermore, we did not evaluate the chest CT features in monitoring the response to therapy.

In conclusion, we think that in case of high clinical suspicion of COVID-19, patients should not be ruled out based on RT-PCR test alone, and the clinical and epidemiologic situation should be carefully considered.

Reduction of lymphocytes, elevation of CRP, LDH, d-dimer, and fibrinogen can be used as an adjunctive diagnostic tool in patient with double negative RT-PCR test and highly suspicious clinic and chest CT scan features.

In addition it is safe to suggest that in emergencies a symptomatic patient with classic CT and lab findings should be quarantined for COVID 19 even with 2 negative RT-PCR tests.

## Author contributions

AO, FG, MM conceived, designed, and wrote the work. SR, ES, DM, EC acquired data. JML, CP, SB analyzed data. MSR provided statistical analysis. AO revised and edited the work.

**Conceptualization:** Antonio Orlacchio, Matteo Santangelo Ravà, Jacopo Maria Legramante, Carla Paganelli, Sergio Bernardini, Marilena Minieri.

**Data curation:** Fulvio Gasparrini, Silvia Roma, Matteo Santangelo Ravà, Daniele Morosetti, Elsa Cossu, Carla Paganelli, Marilena Minieri.

**Formal analysis:** Fulvio Gasparrini, Matteo Santangelo Ravà, Elsa Cossu, Jacopo Maria Legramante, Carla Paganelli, Marilena Minieri.

**Investigation:** Eva Salvatori, Daniele Morosetti.

**Methodology:** Antonio Orlacchio, Jacopo Maria Legramante, Carla Paganelli, Marilena Minieri.

**Resources:** Matteo Santangelo Ravà.

**Software:** Matteo Santangelo Ravà.

**Supervision:** Antonio Orlacchio, Sergio Bernardini, Marilena Minieri.

**Validation:** Elsa Cossu, Jacopo Maria Legramante, Carla Paganelli, Sergio Bernardini.

**Visualization:** Silvia Roma, Eva Salvatori, Daniele Morosetti.

**Writing – original draft:** Antonio Orlacchio, Fulvio Gasparrini.

**Writing – review & editing:** Antonio Orlacchio, Fulvio Gasparrini, Silvia Roma, Sergio Bernardini, Marilena Minieri.
